# Population pharmacokinetic/pharmacodynamic analysis of AK111, an IL-17A monoclonal antibody, in subjects with moderate-to-severe plaque psoriasis

**DOI:** 10.3389/fphar.2022.966176

**Published:** 2022-08-16

**Authors:** Qian Li, Ju Qiao, Hongzhong Jin, Benchao Chen, Zhimei He, Guoqin Wang, Xiang Ni, Max Wang, Michelle Xia, Baiyong Li, Rui Chen, Pei Hu

**Affiliations:** ^1^ Clinical Pharmacology Research Center, Peking Union Medical College Hospital, State Key Laboratory of Complex Severe and Rare Diseases, NMPA Key Laboratory for Clinical Research and Evaluation of Drug, Beijing Key Laboratory of Clinical PK and PD Investigation for Innovative Drugs, Chinese Academy of Medical Sciences and Peking Union Medical College, Beijing, China; ^2^ Department of Dermatology, State Key Laboratory of Complex Severe and Rare Diseases, Peking Union Medical College Hospital, Chinese Academy of Medical Science and Peking Union Medical College, National Clinical Research Center for Dermatologic and Immunologic Diseases, Beijing, China; ^3^ Akeso Biopharma, Inc, Zhongshan, China

**Keywords:** moderate-to-severe plaque psoriasis, psoriasis area and severity index, population pharmacokinetic, population pharmacodynamics, model, monoclonal antibody, IL-17A

## Abstract

AK111 is an innovative IL-17A antibody, presenting high affinity to IL-17A and showing similar pharmacokinetic (PK) characteristics to those of typical immunoglobulin (Ig) G1 antibodies. To optimize the dosage regimen for phase 2/3 clinical trials, PK and pharmacodynamics (PD) of AK111 were first characterized in Chinese moderate-to-severe plaque psoriasis patients in a phase 1b study. AK111 PK serum sample and Psoriasis Area and Severity Index (PASI) score data were collected from 48 moderate-to-severe psoriasis patients in this study. Non-linear mixed-effects modeling was used for the population PK/PD analysis. A one-compartment model with a first-order absorption and a first-order elimination best described the PK behavior of AK111. The apparent systemic clearance was 0.182 L/day, and the central volume was 6.65 L. The exposure–response relationship was characterized using an indirect response model. The pharmacological effect of AK111 was described in the form of inhibiting the formation of psoriatic plaque, whereas placebo was quantified in the form of promoting the degradation of psoriatic skin lesions. The maximum effect of drug effect (I_max_) and placebo effect (PLB_max_) was 1 and 0.429, respectively. The rate constant for psoriatic plaque production (K_in_) was 0.474 PASI/day and psoriatic plaque loss (K_out_) was 0.024 day^−1^. The body surface area (BSA) affected by psoriasis was identified as a significant covariate on $K_{out}$ . The simulation results confirmed that all of the predicted PASI90 response rates at week 12 were higher than 60% at 150 and 300 mg dose levels with different regimens and could reach higher than 80% at week 24. We hope this first PK/PD study of AK111 in Chinese moderate-to-severe plaque psoriasis patients will be of help in the further clinical development of AK111 and provide a reference to the dosage optimization for similar antibodies with a long half-life.

## Introduction

Psoriasis is a chronic immune-mediated autoimmune skin disease. Approximately 125 million people worldwide have psoriasis, which is associated with a number of co-morbidities including psoriatic arthritis, cardiometabolic diseases, and depression (Armstrong and Read 2020). Plaque psoriasis is the most common type of psoriasis, accounting for more than 80% of the psoriasis cases. Plaque psoriasis is characterized by patches of erythema covered in a silvery-white scale, resulting from rapid hyperproliferation and dysregulated differentiation of epidermal keratinocytes (Armstrong and Read 2020; Gisondi et al., 2020). The pathogenesis of psoriasis is complex and not fully elucidated. It is reported to originate from an interplay of genetic, environmental, infectious, and lifestyle factors (Girolomoni et al., 2017). The dysregulated crosstalk between keratinocytes and immune cells contributes to the pathogenesis of psoriasis (Gao et al., 2020). Much evidence suggests that the interleukin (IL)-17/IL-23 axis is a major player in the pathogenesis of psoriasis (Di Cesare et al., 2009), and several biologics targeting the IL-17/IL-23 axis were approved for the treatment of psoriatic diseases (Bai et al., 2019).

AK111 is a humanized IgG1 monoclonal antibody (mAb) that specifically binds IL-17A with high affinity, which is currently in the early stages of clinical research. Cell level *in vitro* pharmacological activity showed that AK111 can block pro-inflammatory cytokine secretion mediated by the IL-17/IL-17 receptor signaling pathway. An *in vivo* efficacy study demonstrated that AK111 could inhibit epidermal thickening, keratinocyte hyperproliferation in mice models of psoriasis (in-house data). A randomized, double-blind, placebo-controlled phase Ib clinical trial of AK111 injection in patients with a moderate-to-severe plaque psoriasis had recently been accomplished. The primary endpoint was to evaluate the safety, tolerability, and pharmacokinetic (PK) profiles of a subcutaneous injection of AK111, and the secondary endpoint was to evaluate its clinical efficacy and immunogenicity.

A reasonable dosing regimen design was one of the key considerations for the next stage’s clinical trial design, establishing exposure–effect relationships based on early PK and clinical efficacy data that can help inform the dose selection. The Psoriasis Area and Severity Index (PASI) is a measure of the average redness, thickness, and scaliness of the lesions weighted by the area of involvement which lies in the range between 0 (no disease) and 72 (maximal disease). It is the current gold standard for the assessment of extensive psoriasis and was employed as the scoring system to assess the efficacy of AK111 in its early phase (Zurauskas et al., 2020). Clinical improvement was measured by the percent change in the PASI score from baseline (Medical Advisory Secretariat, 2009). These time courses of PK and PASI score data collected from the phase Ib study permit an assessment of the exposure–response relationship of AK111 in moderate-to-severe plaque psoriasis.

Population PK-pharmacodynamic (PD) modeling, which links the concentration time course (including variation across dosing intervals) to the time course of the pharmacodynamic response, provides quantitative information to predict the drug exposure–response relationship (Tuntland et al., 2014; Overgaard et al., 2015). It is now well considered that the population PK-PD modeling and simulation are effective tools to determine the optimal dosage and administration frequency of a new drug (Bellissant and Giudicelli, 2001). The overall aim of this study was to develop a population PK/PD model of AK111 to perform a covariate analysis to explain interpatient PK/PD variability. Simulations of different dosing regimens are subsequently performed via this model in order to provide information for the dose regimen selection of the next stage of clinical development.

## Materials and methods

### Study design

The phase I b study was a single-center, randomized, double-blind, placebo-controlled clinical trial, which was conducted in Chinese moderate-to-severe plaque psoriasis patients to observe the pharmacokinetic profile, safety, and efficacy of AK111. The study was conducted in accordance with the Declaration of Helsinki and approved by the independent ethics committee of Peking Union Medical College Hospital (Beijing, China). All patients were fully informed and signed written informed consent forms prior to their enrollment.

Forty-eight patients with moderate-to-severe psoriasis were recruited in accordance with the inclusion and exclusion criteria. They were assigned to four dose cohorts (75, 150, 300, or 450 mg). In each cohort, 12 patients were randomized in a ratio of 3:1 to receive AK111 (*N* = 9) and matching placebo (*N* = 3), respectively. All injections were given subcutaneously at weeks 0, 1, 4, and 8. The serum samples were collected at pre-dose (D1), 2, 4, 8, 15, 22, 29, 36, 57, 85, 113, and 141 days, and postdose-6h at day 1, day 8, day 29, and day 57 to obtain free AK111 concentration. Serum concentrations of AK111 were measured by sandwich enzyme-linked immunosorbent assay (ELISA), the method was fully validated with the lower limit of quantification (LLOQ) being 0.05 ug/mL. PASI scores were evaluated at the time of pretreatment on D8, D15, D29, D57, D85, D113, and D141. Participants who withdraw prematurely were asked to complete serum sample collection and the PASI score assessment at the point of early termination.

### Population pharmacokinetic/pharmacodynamics model building

The population PK/PD model was developed using non-linear mixed-effects modeling [NONMEM v7.3.0, Pirana^®^ v2.9.7, PsN (Perl-speaks-NONMEM) v5.0.0] and visualized using R (v4.0.3)]. A sequential modeling strategy was applied and individual post-hoc Bayesian estimates of the developed PK model were added to the PD dataset. First-order conditional estimation–extension with the interaction (FOCE-I) method was used to estimate the population PK/PD model parameters.

An exploratory graphical analysis was conducted by plotting individual and mean profiles to identify possible starting models and facilitate the identification of potential outliers. One- and two-compartment disposition models were evaluated for the PK, as well as several different absorption models, including first- and zero-order absorption models. Based on the mechanism of action, an indirect response model was chosen as a PD model for the PASI score. Outliers were considered the absolute value of conditional weighted residual (CWRES) greater than 6. They were excluded from our data if these outliers had a negative influence on the convergence and/or made poor estimation precision of parameters, otherwise they will be retained.

An exponential model was used to describe the interindividual variability (IIV):

$$\theta_{i}=\Theta \cdot \text{exp}\left(\eta_{i}\right),$$ (1)

where $\theta_{i}$ represents the parameter estimate for the *i*th individual, θ represents the population estimate for parameter θ , and exp ( $\eta_{i}$ ) is the interindividual random deviation of $\theta_{i}$ from θ *.* $\eta_{i}$ was assumed to be normally distributed with mean zero and variance of $\omega_{i}^{2}$ . As for the residual error model, both additive and multiplicative with the proportional error model were evaluated.

Several variables like age, gender, weight, disease duration, baseline PASI score, body surface area (BSA) involvement, and use of biologics, were tested as potential covariates. A covariate model building was a stepwise process consisting of a forward and a backward selection procedure, in which, continuous covariates were described using the power function and categorical covariates were modeled by an exponential function, see Eqs 2, 3. During the forward inclusion step, a covariate was considered significant and included in the basic model if the OFV decreased by more than 6.635 at the statistical level of *p* value of *<*0.01 (χ2 distribution with 1 degree of freedom). All of the significant covariates were then incorporated into the basic model to construct a full model. Next, backward elimination was used to exclude covariates from the full model with an increase in the OFV of < 10.828 with a *p* value of < 0.001 (χ2 distribution with 1 degree of freedom).

$$\theta_{i}=\Theta \cdot {\left(\frac{cov_{i}}{cov_{median}}\right)}^{\theta_{x}},$$ (2)

$$\theta_{i}=\Theta \cdot {\left(e\right)}^{\theta_{x}*cov}.$$ (3)

Model comparison were based on the objective function value (OFV) using the likelihood ratio test (LRT) for nested models and the Akaike Information Criterion (AIC) for non-nested models. The accuracy of model parameter estimation and the complexity of the model are also factors to be considered when selecting the final model.

### Model evaluation and simulation

The models were evaluated using internal validation techniques. Goodness-of-fit (GOF) plots were used to evaluate the general parameter precision, including conditional weighted residuals (CWRES) versus time, CWRES versus population prediction (PRED), dependent variable (DV) versus PRED or individual population prediction (IPRED). Prediction-corrected visual predictive checks (pvVPC) with 1,000 simulated datasets were also used to assess the predictive performance of the model. In pcVPC, the variability coming from variations in independent variables within a single bin is removed by normalizing the observed and simulated dependent variable based on the typical population prediction (Bergstrand et al., 2011). Results from the pcVPC were assessed by a graphical comparison of the medians and 90% prediction intervals calculated at each bin from the predicted–corrected simulated concentration compared to the observed concentration from the original dataset. A non-parametric bootstrap was performed from which the precision in the parameter estimates were evaluated by the calculation of standard errors.

The final population PK/PD model was used to simulate the PASI score of AK111 after different dosing regimens using a Monte Carlo simulation approach. The model was run with 1,000 Monte Carlo simulations using typical values and interpatient variability estimated from the final PK/PD model, the covariates of the virtual patient still leveraged those of the phase Ⅰb dataset. The PASI75 and PASI90 (75% and 90% reductions in the PASI score from the baseline) response rates were calculated using the PASI score predicted by the model.

## Results

### Population characteristics

In total, 48 moderate-to-severe plaque psoriasis patients were enrolled, wherein 12 patients received placebo and 36 patients received AK111 injection. Baseline demographics and subject characteristics of the 48 patients are summarized in Table 1. One subject in the 450 mg cohort was not included in the analysis due to delayed administration, and 47 patients were available for the PK/PD analysis. Finally, a total of 516 PK samples from patients treated with AK111 and 344 PASI scores from patients treated with AK111 and placebo were used for PK/PD modeling, and no outliers were found in our PK/PD data. Concentrations below the LLOQ (< 6%) were only distributed at time zero and were excluded according to the M1 method recommended by Keizer et al. during PK model building (Keizer et al., 2015). All PASI score data were included in the final PK/PD model building.

$$\frac{dA_{depot}}{dt}=-Ka*A_{depot}\left(t=0,A_{depot}=0\right),$$ (4)

$$\frac{dA1}{dt}=Ka*A_{depot}-CL*C\left(t=0,A1=0\right),$$ (5)

$$\frac{dPASI}{dt}=K_{in}\times \left(1-\frac{I_{max}\times C}{IC_{50}+C}\right)-K_{out}\times PLB\times PASI,$$ (6)

$$K_{out}=tvK_{out}*{\left(\frac{BSA}{31}\right)}^{\theta BSA}*exp\left(nK_{out}\right),$$ (7)

$$\text{PLB}=1+PLB_{max}*e^{-Kplb*t}.$$ (8)

**TABLE 1:** Demographic and baseline characteristics (mean ± SD).

Characteristic	75 mg	150 mg	300 mg	450 mg	Placebo	Total
Number of subjects	9	9	9	9	12	48
Age (years)	33.4 ± 6.9	41.7 ± 11.2	39.0 ± 7.9	38.3 ± 9.1	38.7 ± 8.4	38.2 ± 8.9
Sex-female, n (%)	2 (22.2%)	2 (22.2%)	2 (22.2%)	2 (22.2%)	2 (16.7%)	10 (21.3%)
Height (cm)	166.9 ± 6.5	163.8 ± 6.1	170.0 ± 8.3	169.9 ± 9.2	167.7 ± 9.3	168 ± 8.1
Weight (kg)	65.7 ± 10.1	64.2 ± 8.7	71.0 ± 8.8	67.3 ± 10.3	69.2 ± 11.3	67.5 ± 10.0
BMI (kg/m2)	23.5 ± 3.0	23.9 ± 2.4	24.5 ± 1.4	23.3 ± 3.0	24.5 ± 2.9	24 ± 2.6
Disease duration (years)	14.8 ± 5.4	16.3 ± 5.0	18.3 ± 5.7	12.8 ± 6.3	15.8 ± 4.9	15.7 ± 5.4
BSA (%)	30.6 ± 12.3	30.8 ± 15.2	38.7 ± 16.4	36.5 ± 18.7	30.8 ± 11.2	33.3 ± 14.7
Baseline PASI	19.1 ± 4.7	16.9 ± 3.8	22.8 ± 6.4	22.8 ± 7.4	18.5 ± 3.4	20 ± 5.6
Use of biologicals, n (%)	0 (0.0%)	2 (22.2%)	1 (11.1%)	1 (11.1%)	0 (0.0%)	4 (8.5%)

### Population pharmacokinetic/pharmacodynamics model building

**FIGURE 1:**
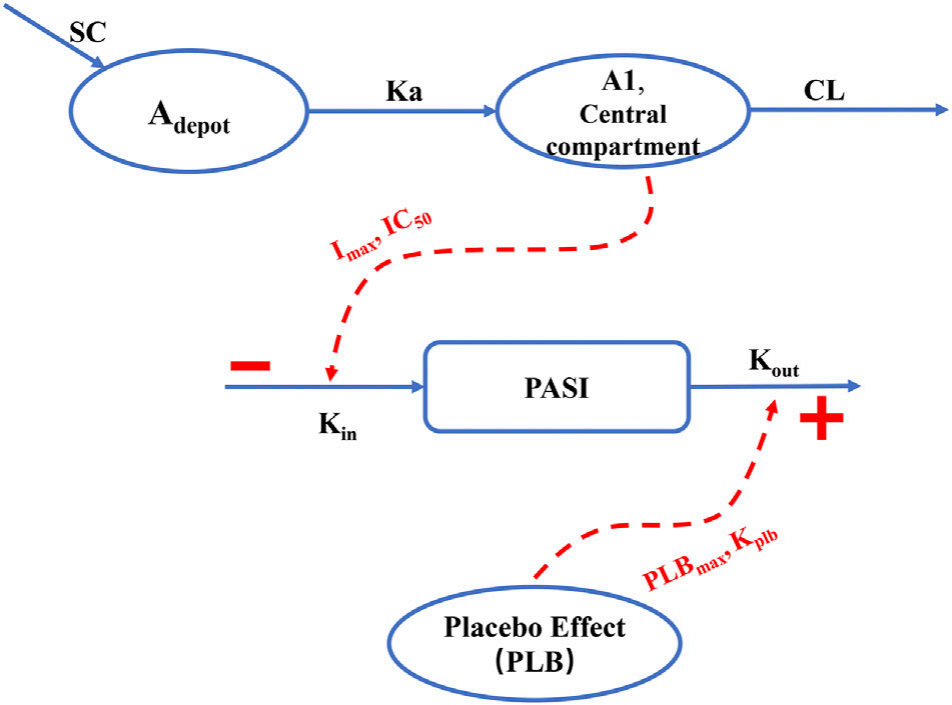
Schematic overview of the final population PK/PD model. SC: subcutaneous injection; A_depot_: AK111 amounts in absorption depot; A1: AK111 amounts in central compartment; Ka: subcutaneous absorption rate constant; CL: clearance from the central compartment $K_{in}$ : zero-order formation rate constant for the psoriatic plaques; $K_{out}$ : first-order degradation rate constant for the psoriatic plaques; I_max_: maximum inhibition effect on plaque forming rate constant; IC_50_: concentration at half-maximal inhibition; $PLB_{max}:$ maximum effect of placebo effect; $K_{plb}$ : rate constant of the reduction from the maximum placebo effect.

According to the AIC value and the GOF plots, the selected final proposed PK/PD model structure is shown in Figure 1. A one-compartment with a first-order absorption and a first-order elimination model best described the PK behavior of AK111. No covariates were found to be associated with the PK parameters. The PASI score was fitted well through an indirect response model with both the drug effect and placebo effect, and BSA was identified as a significant covariate on $K_{out}$ . The model assumed that baseline PASI was at the steady state prior to dosing and was governed by a zero-order formation rate constant ( Kin ) and a first-order degradation rate constant ( Kout ) for the psoriatic plaques. AK111 in the central compartment inhibited Kin to the inhibited plaque-forming process. And, placebo reduced the PASI score by increasing the degradation rate constant ( Kout ) for the psoriatic plaques. All PK/PD model parameters are summarized in Table 2. The mathematical representation of the final model is provided in Eqs 4–8.

**TABLE 2:** The estimated value of the PK/PD parameters in the model.

Parameters (unit)	Definition	Point estimate	RSE%	Bootstrap of estimates median	Bootstrap of estimates 90%CI	Shrinkage%
PK model						
Ka (1/day)	Subcutaneous absorption rate constant	0.463	9.3	0.461	0.392–0.534	NA
CL (L/day)	Clearance from the central compartment	0.182	7.2	0.182	0.159–0.205	NA
V (L)	Volume of the peripheral compartment	6.65	7.8	6.65	5.77–7.54	NA
IIV Ka (%)	Interindividual variability in Ka	50.1	13.5	48.9	36.6–60.4	5.7
IIV CL (%)	Interindividual variability in CL	42.2	12.4	41.5	33.0–49.5	0.1
IIV V (%)	Interindividual variability in V	46.4	13.4	45.6	35.1–54.6	0.1
σ_prop_	Proportional error for serum concentration	0.0144	9.4	0.0146	0.012–0.017	10
σ_addi_	Additive error for serum concentration	1.48	42.4	1.372	0.442–2.51	10
PD model						
K_in_ (PASI/day)	First-order rate constant for psoriatic plaque production	0.474	21.3	0.481	0.305–0.643	NA
K_out_ (1/day)	Zero-order rate constant for psoriatic plaque loss	0.024	21.5	0.025	0.015–0.033	NA
I_max_	The maximum effect of drug effect	1	Fixed	1	Fixed	NA
IC_50_ (ug/mL)	The concentration to achieve 50% Emax	0.52	66.4	0.566	0–1.174	NA
PLB_max_	The maximum effect of placebo effect	0.429	78.2	0.394	0–1.056	NA
K_plb_	Rate constant of reduction from maximum effect	0	Fixed	0	Fixed	NA
θBSA	Covariate about BSA on Kout	−0.572	−7.68	−0.573	−0.644—0.499	NA
IIV K_in_ (%)	Interindividual variability in Kin	16.0	38.7	16.8	5.90–25.2	98.8
IIV K_out_ (%)	Interindividual variability in Kout	23.3	13.8	22.2	15.5–27.5	18.3
IIV IC_50_ (%)	Interindividual variability in IC50	161.2	33.5	161.2	67.6–449.6	47.8
IIV PLB_max_ (%)	Interindividual variability in PLBmax	98.7	15.0	96.4	64.7–138.2	20.8
σ_PASI_	Additive error for PASI score	4.53	21.6	4.49	2.92–6.13	11.5

RSE, relative standard error; CI, confidence interval.

### Model evaluation

**FIGURE 2:**
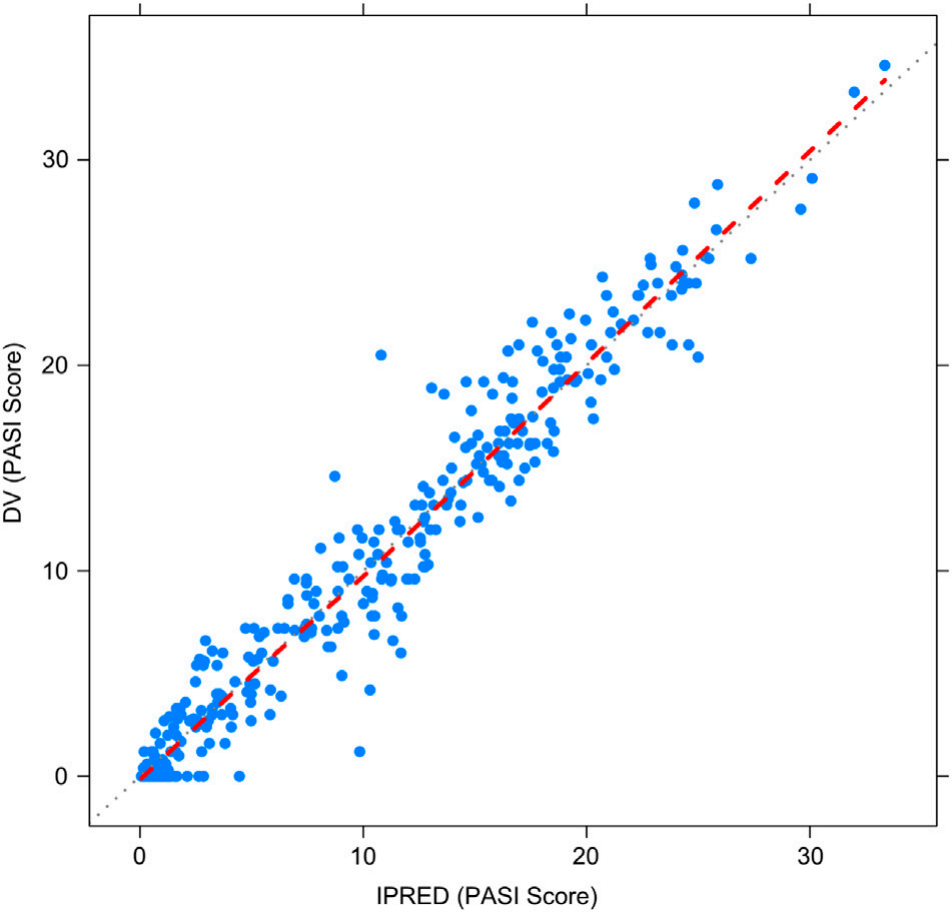
Goodness-of-fit plots for the final population PK/PD model. Observed PASI score (DV) vs. Individual-predicted PASI score (IPRED).

**FIGURE 3:**
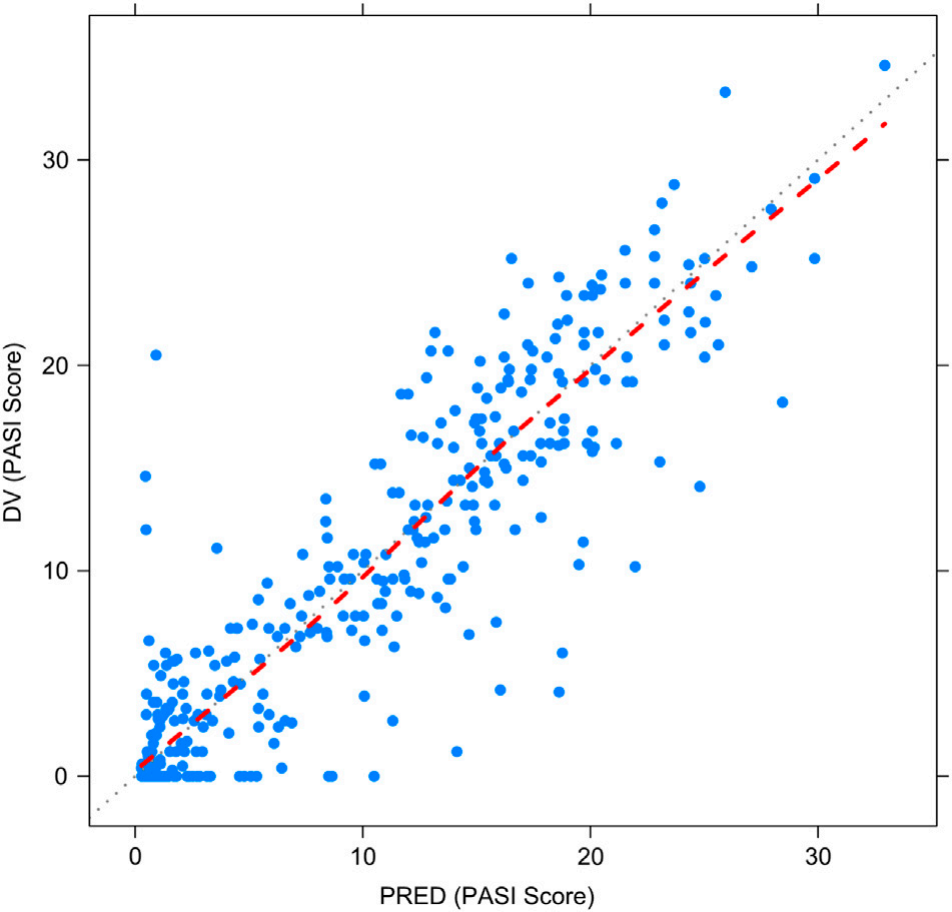
Goodness-of-fit plots for the final population PK/PD model. Observed PASI score (DV) vs. Population-predicted PASI score (PRED).

**FIGURE 4:**
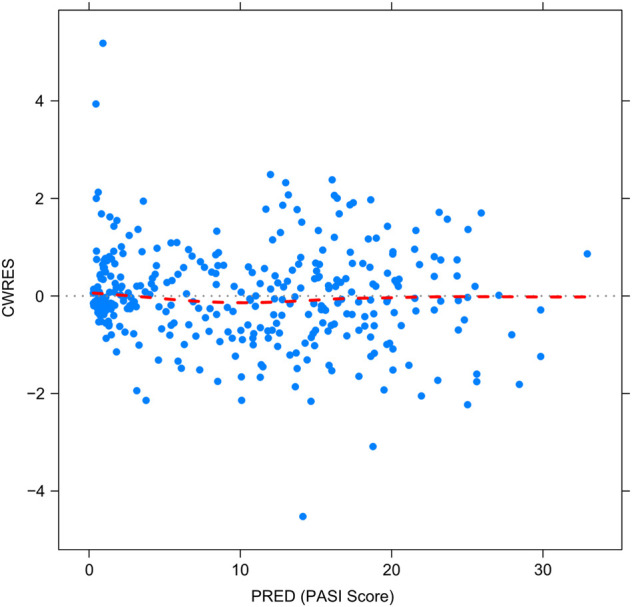
Goodness-of-fit plots for the final population PK/PD model. The conditional weighted residuals (CWRES) vs. population-predicted PASI score (PRED).

**FIGURE 5:**
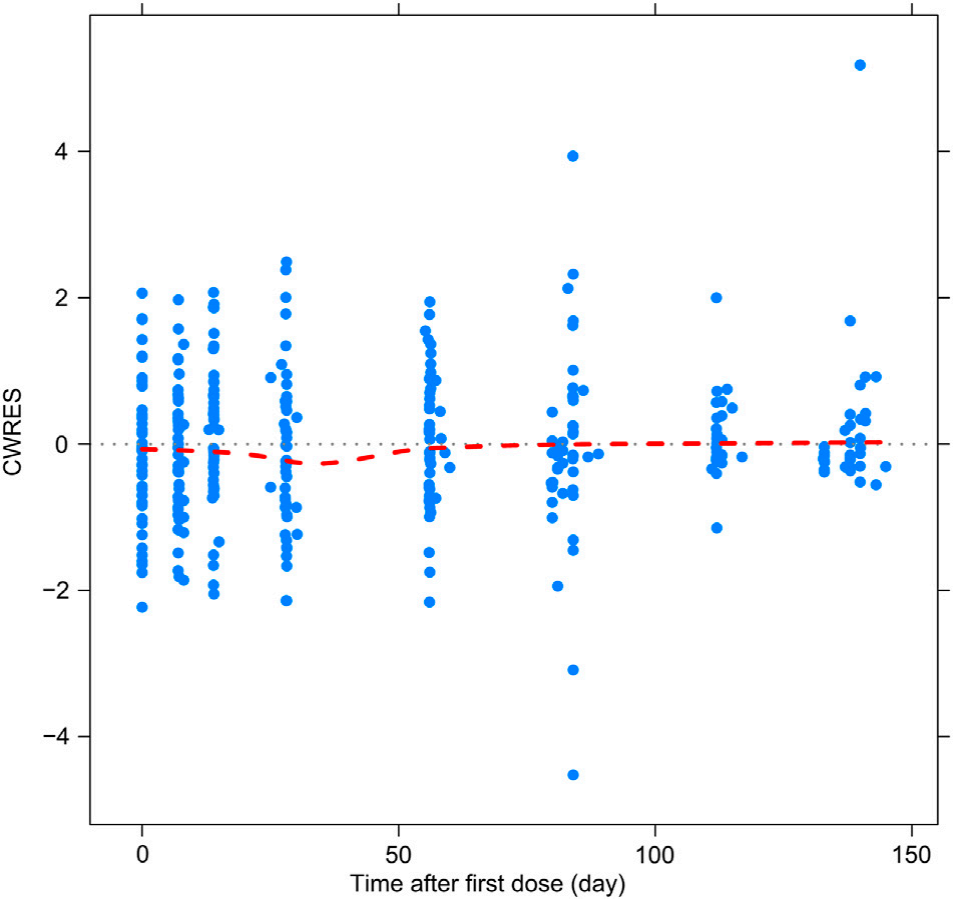
Goodness-of-fit plots for the final population PK/PD model. CWRES vs. time after first dose.

Figures 2–5 show the GOF plots of the final population PK/PD model. Plots of the dependent variable (DV) versus population prediction (PRED) or individual population prediction (IPRED) demonstrated that the final model fits the data well. There were no obvious deviations in the plots of conditionally weighted residuals (CWRES) vs. time or population prediction (PRED). The plots were symmetrically distributed about the zero axis and most points were laid within the acceptable range (−1.96 to 1.96).

**FIGURE 6:**
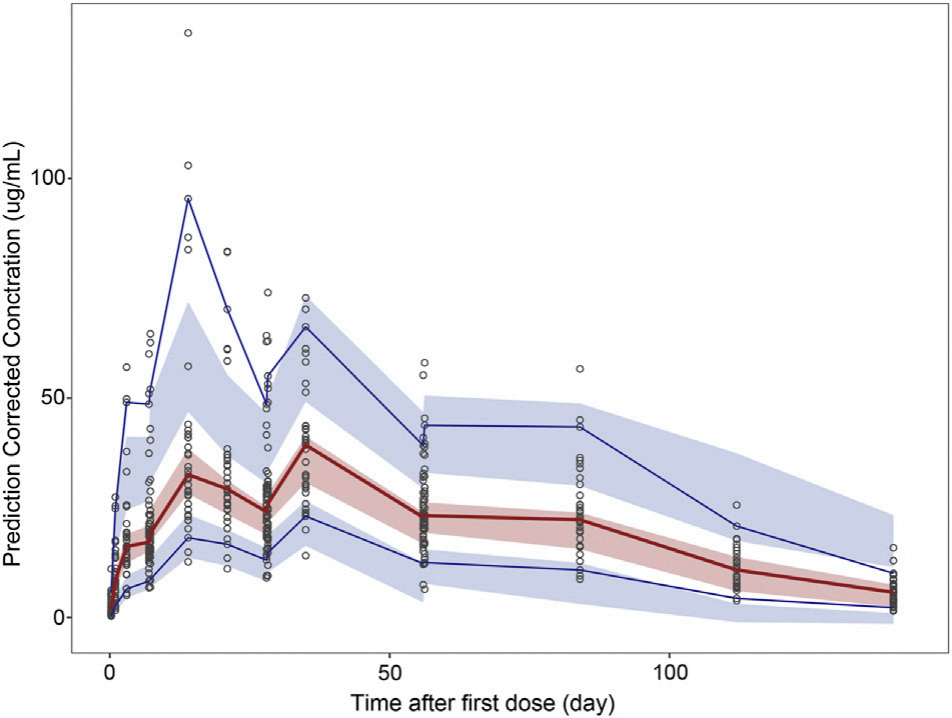
Prediction-corrected visual predictive check (pcVPC) results for population PK and PK/PD models. pcVPC from the population PK model.

**FIGURE 7:**
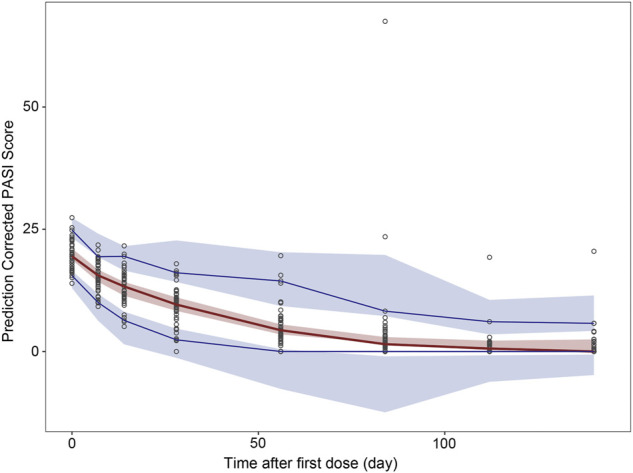
Prediction-corrected visual predictive check (pcVPC) results for population PK and PK/PD model. pcVPC from final population PK/PD model. Black circles represent prediction-corrected observation points. Red solid lines represent the 50th percentiles and blue solid lines represent the 5th and 95th percentiles of prediction-corrected observations; Red shadows around the curves represent 90% confidence intervals of the 50th percentiles of prediction-corrected prediction and blue shadows represent 90% confidence intervals of the 5th and 95th percentiles for prediction-corrected prediction.

The pcVPC method could remove the variability coming from binning across an independent variable by normalizing the observed and simulated dependent variable, while retaining the visual interpretation of the traditional VPC (Bergstrand et al., 2011). The pcVPC results for the population PK and PK/PD models are shown in Figure 6 and Figure 7. The pcVPC from the population PK model confirmed the robustness of the model stability and PK parameters were estimated with good precision with 90% of the observed values within the 5th and 95th percentiles of the simulated data (Figure 6). One observed concentration at day 22 was significantly higher than the others, resulting in a lower prediction of the 95th percentile of observation. The pvVPC of the PK model also showed a slight underprediction around the 5th percentile of observation after day 113. But they have little impact on the clinical utility of this model because most of the 5th, 50th, and 95th percentiles of prediction were in good agreement with the observed data.

The pcVPC of the final PK/PD model showed that the 50th and 95th percentiles of the prediction-corrected prediction PASI scores were consistent with the 50th and 95th percentiles of observations, even though the 5th percentile was slightly under-predicted (Figure 7). As treatment progresses, the predicted 5th percentile of PASI becomes out of data range (< 0), this is because there was great interindividual variation in the PASI score, especially those time points after 84 days. When the predicted PASI score became near 0, together with high variation and residual errors, this led to 5th percentile of PASI score below zero. They did not impact the clinical utility of this model because the predicted PASI score below zero meant the psoriasis plaque was complete eliminated, the scores were treated as zero when calculating the response rate. Despite these deviations, the 90% prediction interval of the simulated data covered most of the observations. The bootstrap results are also shown in Table 2. The median values from the bootstraps were close to the typical values of parameter estimates of the final model. All the typical parameter values fell within 90 % CI of the bootstrap results, which indicated the high stability and precision of the final model.

### Model simulation

**FIGURE 8:**
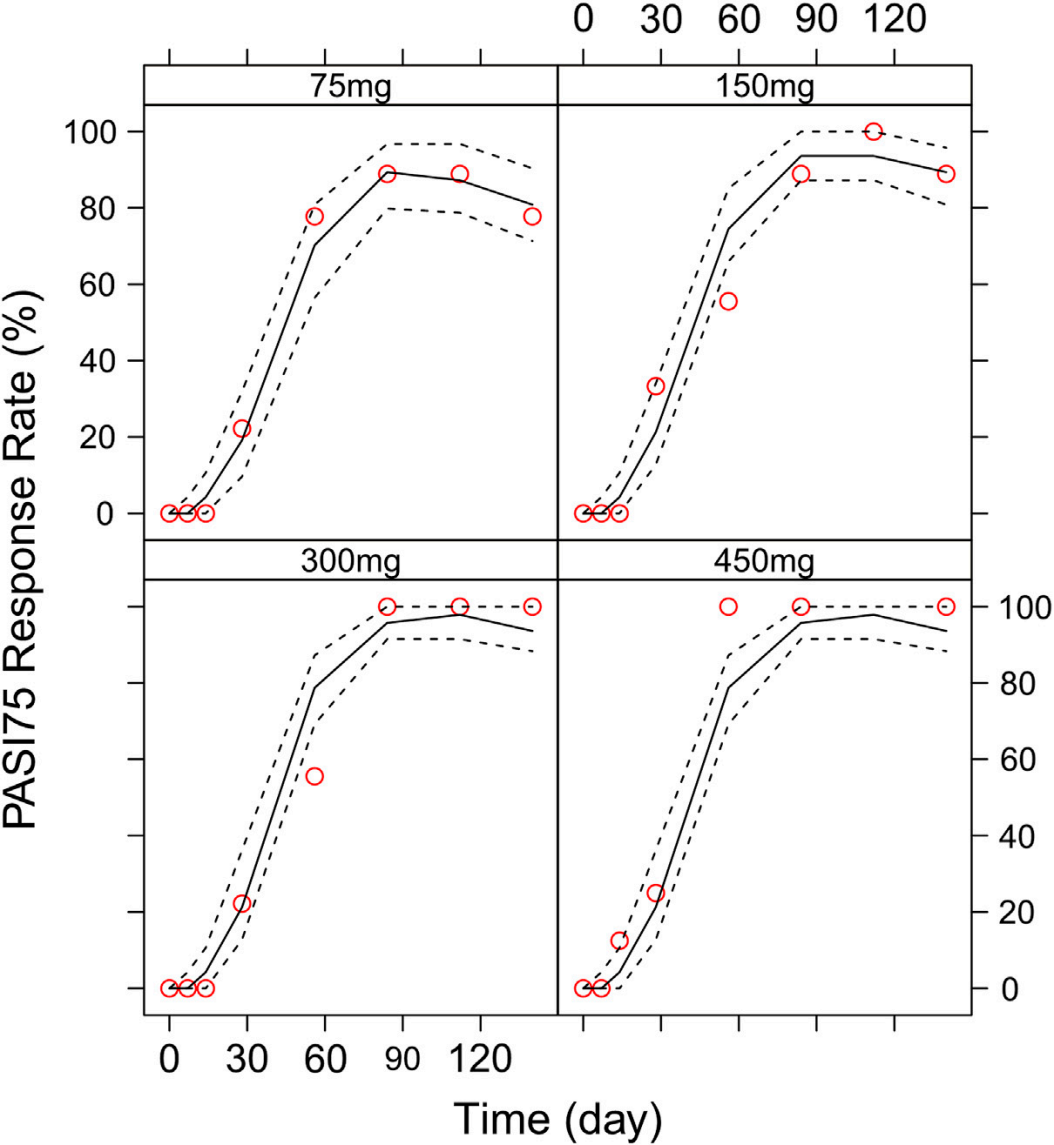
Comparison of the observed time profiles of PASI75 and PASI90 response rates versus model-predicted time profiles stratified by dose. Observed and predicted PASI75 response rates stratified by dose.

**FIGURE 9:**
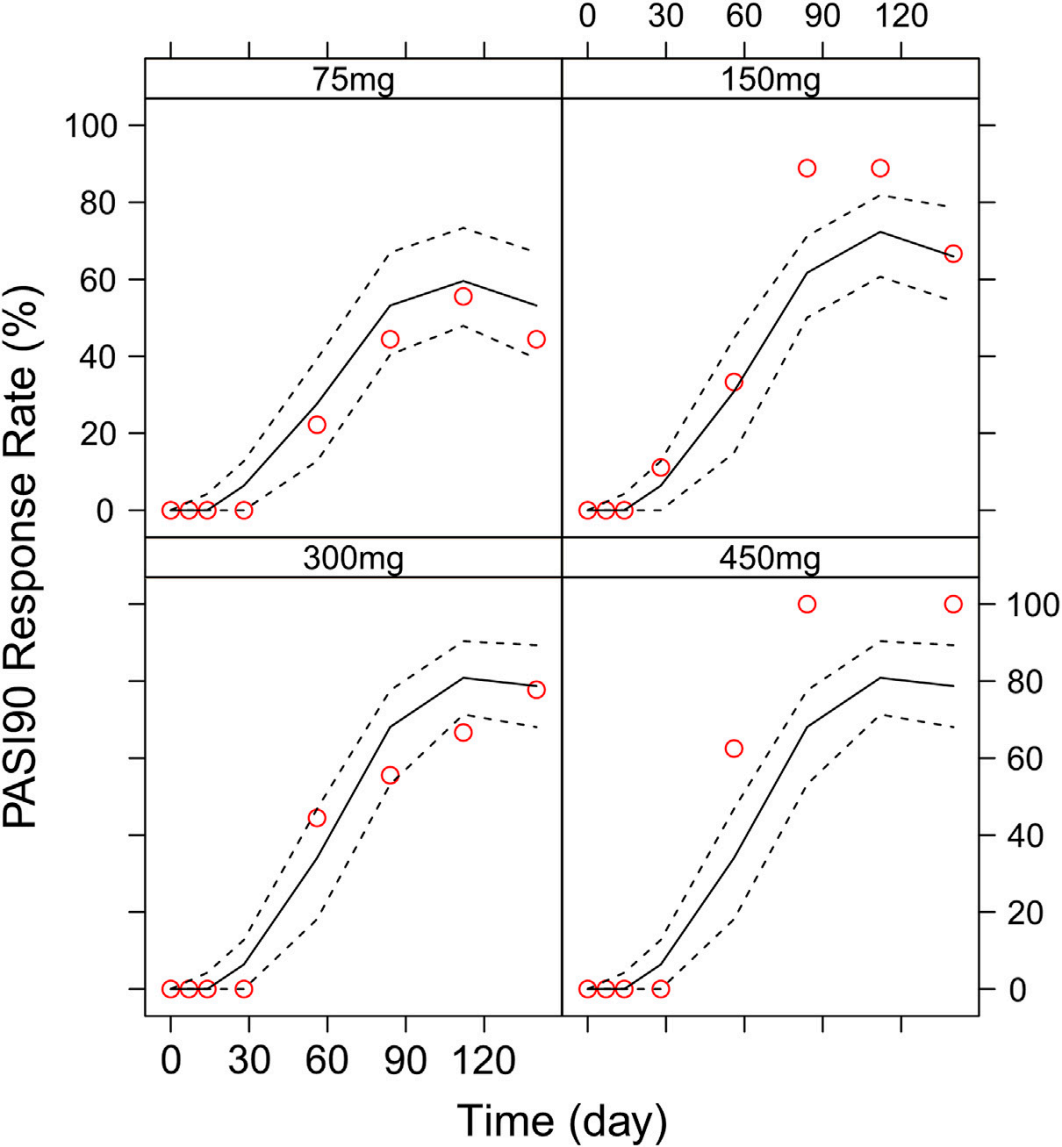
Comparison of the observed time profiles of the PASI75 and PASI90 response rates versus model-predicted time profiles stratified by dose. Observed and predicted PASI90 response rates stratified by dose. Red circles represent observed response rates; black lines represent the 50th percentile of the predicted response rate; the two black-dotted lines represent a 95 % confidence interval for the predicted response rate.

Different dosing regimens of AK111 were simulated using Monte Carlo methods to obtain PASI scores, and the PASI75 and PASI90 response rates were calculated. Figure 8 and Figure 9 shows the comparison time profiles of the PASI75 and PASI90 response rates observed versus model-predicted time profiles stratified by dosage. The values of the PASI75 response rates observed at various time points were almost within 95% prediction intervals in the four dose levels (Figure 8). The predicted PASI90 response rate of 450 mg dose level was lower than the observation after day 29 (Figure 9). The main reason for the underprediction of the PASI90 response rate in these dose levels could be that one subject was excluded due to delayed administration; there were only eight subjects in this cohort. A more detailed explanation can be found in the Discussion.

**FIGURE 10:**
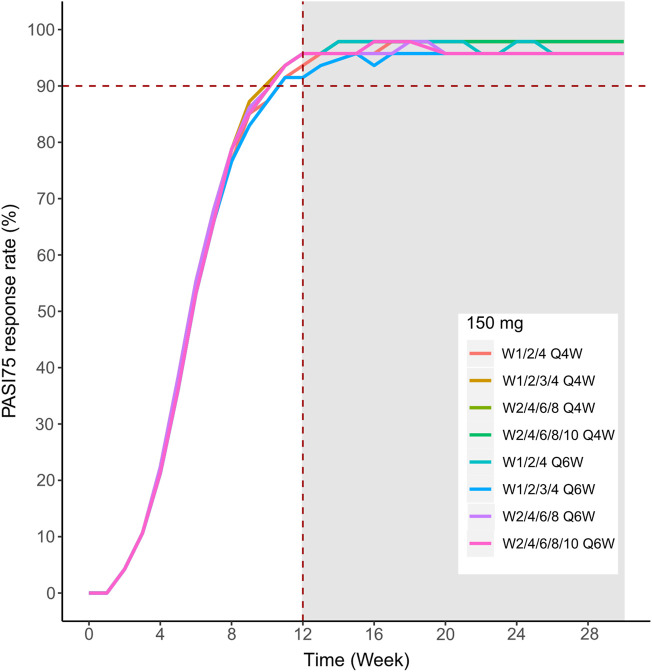
Simulated median PASI75 and PASI90 response rates under different dose regimens using the final population PK/PD model. PASI75 response rate of 150 mg in the eight dosing regimens.

**FIGURE 11:**
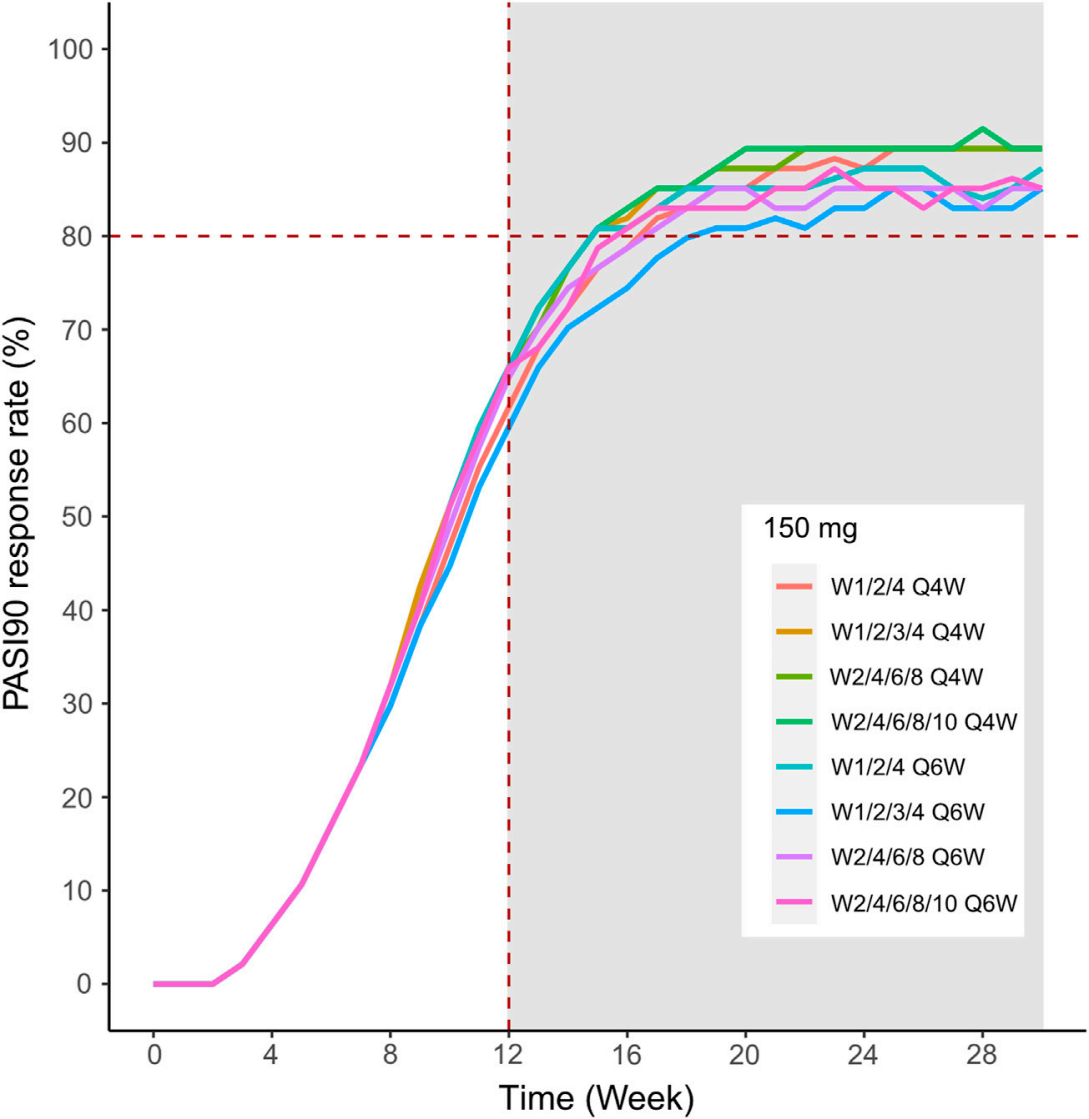
Simulated median PASI75 and PASI90 response rates under different dose regimens using the final population PK/PD model. The PASI90 response rate of 300 mg in eight dosing regimens. Different colors of lines represent the median response rate of different dosing regimens.

**FIGURE 12:**
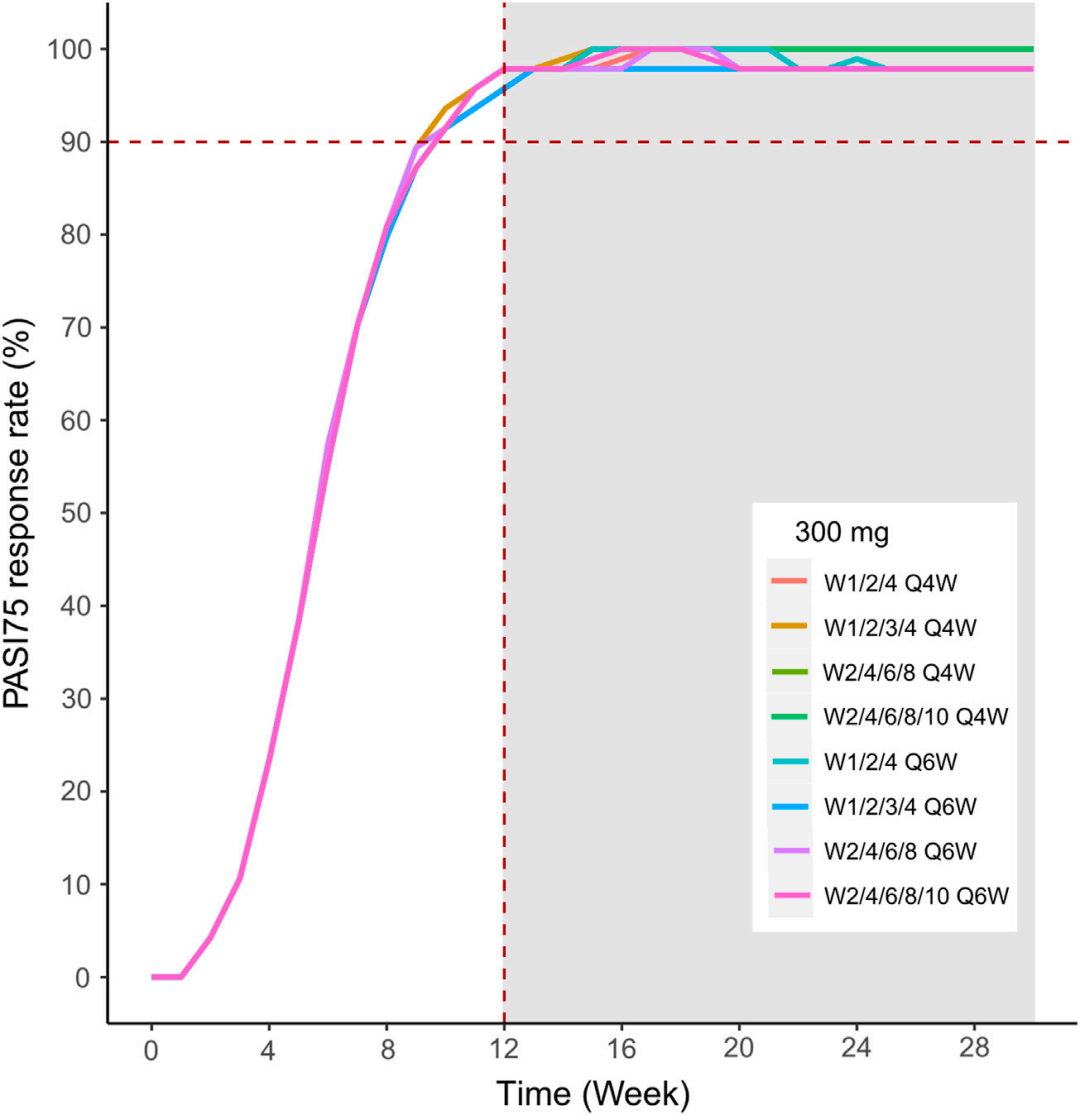
Simulated median PASI75 and PASI90 response rates under different dose regimens using the final population PK/PD model. The PASI75 response rate of 300 mg in eight dosing regimens.

**FIGURE 13:**
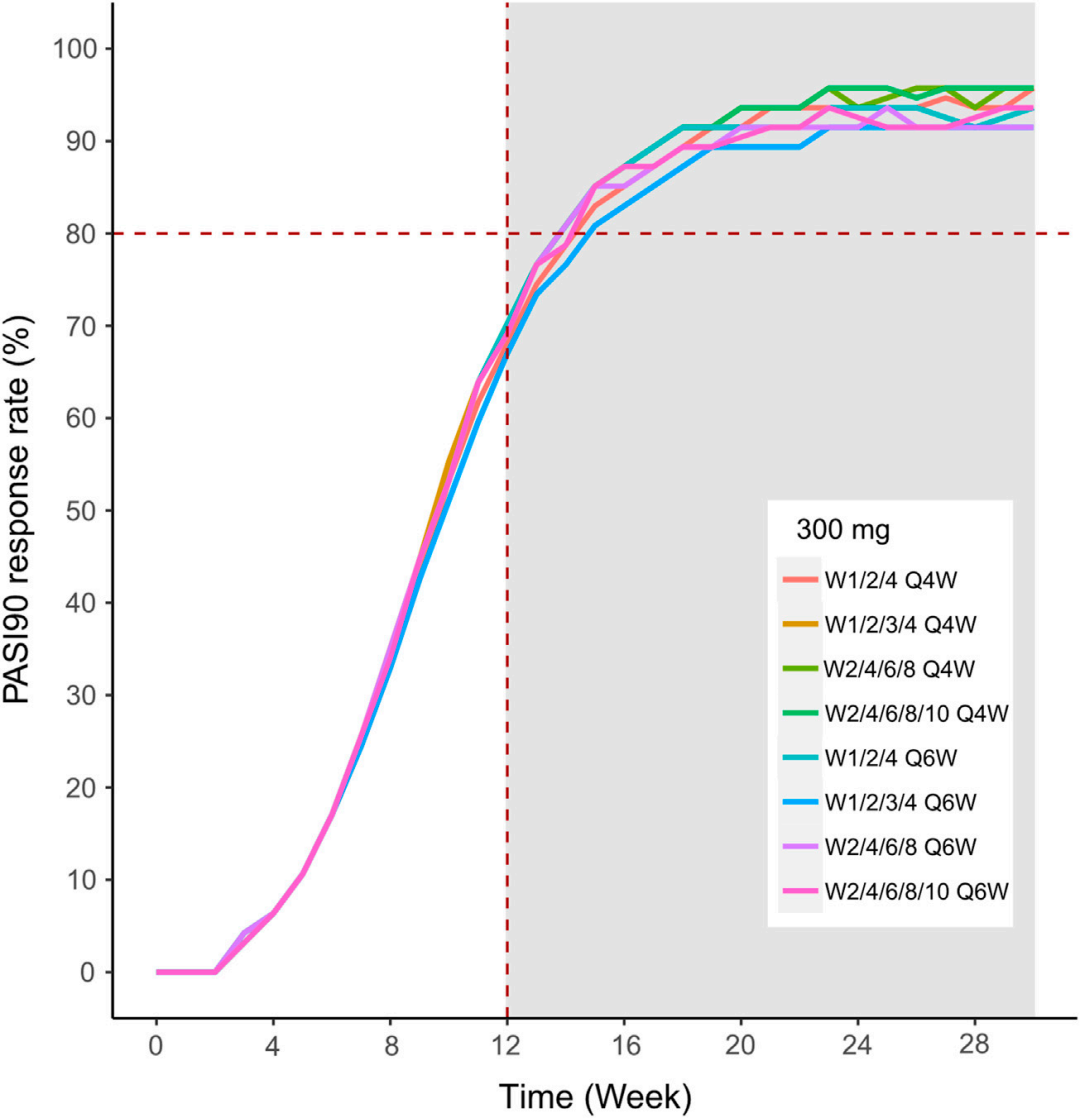
Simulated median PASI75 and PASI90 response rates under different dose regimens using the final population PK/PD model PASI90 response rate of 150 mg in eight dosing regimens.

We then simulated the 150 and 300 mg dose levels with different loading and maintenance dose frequencies: 1) dose at weeks 0,1, and 4, followed by Q4W or Q6W; 2) dose at weeks 0, 1, 2, 3, and 4, followed by Q4W or Q6W; 3) dose at weeks 0, 2, 4, 6, and 8, followed by Q4W or Q6W; 4) dose at weeks 0, 2, 4,6, 8, and 10, followed by Q4W or Q6W. The predicted medians of the PASI75 and PASI90 response rates of the eight dosing regimens are showed in Figures 10–13. The simulation results suggested that for each dose level, at the initial phase using different loading dosing regimens, the onset time did not appear to change significantly, this result needs to be verified by sufficient observational data. Compared with Q4W, the administration of Q6W showed similar PASI75 and PASI90 response rates in the maintenance period. At week 12, the predicted median PASI75 response rate in each dose regimen was above 90%, and was close to 100% till week 24 (Figure 10 and Figure 12). All of the PASI90 response rates at week 12 were higher than 60% at 150 and 300 mg dose levels with different regimens, and could reach higher than 80% at week 24 (Figure 13 and Figure 11).

## Discussion

Population PK and PD modeling of antibodies is instrumental for dosage designing and optimizing throughout the drug development process (Wang et al., 2008). In the present study, a population PK-PD model was established in the Chinese moderate-to-severe plaque psoriasis patients and simulations were performed to guide the AK111 dose selection for phase 2/3 trials.

Although the pathogenesis of psoriasis is still not fully elucidated, molecular and genetic studies have identified the main inflammatory pathways involved in the pathogenesis of psoriasis (Rendon and Schakel 2019). Several mAbs designed to block either specific receptors or soluble mediators of the main pathways were approved for treating psoriasis, including TNF-α, IL-12/23, and IL-17 antibodies (Rodriguez-Fernandez et al., 2022). Secukinumab, ixekizumab, and brodalumab are three anti-IL-17 mAbs which are already available in the market (Mrowietz et al., 2021), AK111 is also targeting IL-17A. Despite the same target site, the PK and PD properties of these molecules are specific. To the best of our knowledge, this is the first study to report the PK and PD properties of AK111 in Chinese moderate-to-severe plaque psoriasis patients.

The PK profile of AK111 was well described by a one-compartment model with first-order absorption. During model building, no target-mediated drug disposition was observed. The data were well fitted by first-order elimination kinetics. A two-compartment model was tried when building the PK model, however, little effect on the improvement of the model was observed, and the estimation accuracy of the parameters was poor and the RSE% values of some parameters were extremely high. The one-compartment model was finally selected considering that the elimination phase of the last administration was not dense sampling and the intercompartmental clearance rate of the two-compartment model may not be well estimated.

Based on the population PK model, the Ka value estimated was low (0.463 day^−1^). These data suggest that AK111 was slowly absorbed from the injection site following the subcutaneous injection (75–450 mg). With the molecular weight of approximately 150 kDa, the extravascular distribution of AK111 is expected to be very small. The apparent volume of distribution in moderate-to-severe psoriasis patients was low (6.65 L), which was slightly greater than the serum volume. The majority of IgG mAbs cannot be filtered by the kidney. Their elimination occurs via intracellular catabolism, including fluid-phase or receptor-mediated endocytosis (Wang et al., 2008). AK111 showed a slow serum clearance (0.182 L/day) with an average elimination half-life (t_1/2_) of 25.3 days ( 0.693*V/CL ). The t_1/2_ is similar with Secukinumab (a human IgG1 anti-IL-17A antibody), and longer than another IL-17A antibody Ixekizumab (t_1/2_ = 13 days) (Craig and Warren 2020). The long half-life could support longer dosing intervals, which can improve the adherence of patients from a clinical perspective, especially for chronic diseases.

The most common clinical covariates for mAbs treating psoriasis were body weight and the presence of immunogenicity on CL (Bruin et al., 2017; Rodriguez-Fernandez et al., 2022). For AK111, none of the covariate (age, gender, weight, disease duration, baseline PASI score, and BSA) relationships were identified in the developed population PK model. The reason was the small size of the sample and a narrow range of continuous covariate distributions. In this case, the value of the covariate analysis was limited, and we used a model with no covariates as the final model.

For the clinical study in psoriasis, the PASI score is typically used for the diagnosis and evaluation of psoriatic diseases (Ashcroft et al., 1999). The score combines the extent of involvement and severity of redness, thickness, and scaling from each body region (head, trunk, arms, and legs) to give a score ranging from 0 to 72 with higher scores indicating greater disease severity (Salinger et al., 2014). Based on an exploratory analysis of our data, we observed that most subjects had achieved the greatest reduction in the PASI scores till 12 weeks after the first dose administration with a delay relative to the peak concentration. The indirect response (IDR) modeling approach capable of characterizing the lag in time between time course of pharmacokinetic and pharmacodynamic events, and could explain the formation and elimination of plaque dynamics semi-mechanistically. This semi-mechanistic pharmacokinetic–pharmacodynamic (PK-PD) models in psoriasis have been published for a few biologic compounds based on the PASI score (Zhou et al., 2010; Salinger et al., 2014). We also tested several other models during PD model building, including an effect-compartment model, effect-compartment link indirect response (IDR) model, logistic regression model (considered the PASI75 response as dichotomic variables), Emax model using the rate of the PASI75 response (response rate from 0% to 100%) as the outcome; however, these models either did not perform better than the IDR model or failed to be established due to limited sample size.

Improvement in the PASI scores was observed in the placebo group. Though the effects were small, they could not be neglected. Thus, the placebo effect was integrated in the model, and the parameters related to placebo were estimated from patients who received placebo. The reported placebo effects either act on formation of the plaques (Salinger et al., 2014) or the degradation of plaques (Tham et al., 2014). In order to avoid a co-mingled drug and placebo effect, we assumed that the placebo reduced the PASI score by increasing the degradation rate constant (K_out_) for the psoriatic plaques.

The drug-related PD parameter estimates from this model were found to be comparable to those reported for another anti-IL-17A monoclonal antibody (Brodalumab) in patients with moderate-to-severe plaque (Salinger et al., 2014). The I_max_ was fixed to 1 because the maximal effect could theoretically be a full 100% reduction in the PASI score which can be seen from our PASI score data, this was the same as the results David H. Salinger reported (Salinger et al., 2014). The concentration to achieve 50% E_max_ (IC_50_) was 0.52 ug/mL with RSE% was 66.4%, and was not associated with any of the covariates. The higher RSE% was also observed in Brodalumab (49.7%) and Ixekizumab (60.3%) (Salinger et al., 2014; Tham et al., 2014), this is because the subpopulations of the responder and non-responder make the distribution of the post hoc IC_50_ parameter as a bimodal distribution (Zhou et al., 2010; Tham et al., 2014; Pan et al., 2020), but in our study, the sample size was too small to distinguish two subpopulations. K_in_ and K_out_ were parameters that related to the disease, the reported K_in_ was 0.615–0.890 PASI/day, K_out_ was 0.0313–0.064 day^−1^ (Zhou et al., 2010; Salinger et al., 2014; Tham et al., 2014; Rodriguez-Fernandez et al., 2022). In our study, K_in_ was 0.474 PASI/day, K_out_ was 0.024 day^−1^, and both of them were lower than those reported in the literature. The possible reason is that the estimation of these two parameters depends on the baseline PASI score, which is different among patients enrolled in different clinical studies.

The maximum placebo effect (PLB_max_) was 0.429 with RSE % being 78.2 %, similar to that reported by David H. Salinger (0.439 with 59.5 % SE) (Salinger et al., 2014). Another placebo-related parameter was the rate constant of reduction from maximum effect (K_plb_), the value reported in the literature was 0.046 day^−1^ (with 49.3%SE) (Salinger et al., 2014). During the progress of modeling, the K_plb_ value estimated in our model was much lower than reported (<10^−4^, data not shown), this indicated that the rate constant of reduction from the maximum effect was negligible, so the K_plb_ value was fixed to zero in our final model.

Among all covariates analyzed of the influence on PD parameters, we found that BSA was the most influential covariate correlating with K_out_, this has not been reported in previous studies. Chuanpu Hu (Hu et al., 2018) reported the body weight effect on K_out_ in guselkumab exposure–response relationship studies, the difference between our result with Chuanpu Hu may be because we used the PASI score as the outcome to established the relationship between exposure and efficacy in our model, but Chuanpu Hu used the PASI Response Thresholds (Prt) in their model, so the K_out_ of the two models has different meanings. Finding the source of interindividual variation of IC_50_ is important because its large IIV and different individuals have different treatment outcomes; higher doses may be required for non-responder subpopulations (Tham et al., 2014). Lai-San Tham reported the PASI75 responder status at week 12 as a significant covariate for the EC_50_ parameter (Tham et al., 2014; Rodriguez-Fernandez et al., 2022), some literatures reported that the covariate analysis identified high-sensitivity C-reactive protein (hs-CRP) as a statistically significant covariate for risankizumab EC_50_ (Khatri et al., 2020; Suleiman et al., 2020). In our analysis, the addition of any covariable does not reduce the IIV of this parameter; one possible reason is that the sample of 47 is too small. The effect of covariates on placebo-related parameters has not been reported in previous studies, due to the smaller sample size (*n* = 12), it is also difficult to explain the interindividual variation in placebo parameters for us.

Different dose regimen simulations based on this population PK/PD model were performed. Based on the observed efficacy of this study, 150 and 300 mg were selected as the candidate dose levels in the next phase II clinical trial, because the curves of mean PASI score over time at both dose levels were close to 450 mg (data not shown) and the maximum PASI75 response rates both could reach the maximum of 100 % (Figure 8). Psoriasis is a chronic disease that requires long-term management, systemic therapy of plaque psoriasis consisting of the induction phase (usually 12 weeks or 16 weeks) and the maintenance phase (Mrowietz et al., 2011). Our result of observed and predicted response rates showed that there needs to be 12 weeks after administration for the maximal response rate. So, another concern was if the initial phase using loading dosing regimens could allow the patient to achieve maximum efficacy quickly. So, we designed four loading dosing regimens (W1/2/4; W1/2/3/4; W2/4/6/8; and W2/4/6/8/10) to be simulated. To maintain the long-term efficacy of AK111, rational administration frequency needed to be studied. We simulated the dosing frequency for Q4W based on the half-life of AK111 (t_1/2_ = 25.3 days), a longer dosing interval Q6W was also simulated and compared with Q4W.

The PASI75 and PASI90 response rates of different dosing regimens were calculated based on the PASI score simulated by the final PK/PD model. In the past, the treatment goal of psoriasis was the PASI75 response proposed by the European Consensus Programme (ECP) (Mrowietz, 2012) and FDA also uses PASI75 as a primary efficacy endpoint for new psoriasis drugs. Now, there is an accumulation of evidence in support of the PASI90 response becoming the new therapeutic goal standard based on its better correlation with health-related quality of life (HRQoL) improvement (Puig, 2015). Several models have previously been reported in the literature using categorical scores such as PASI75 as an outcome indicator to establish the population pharmacokinetic–logistic regression model (Hutmacher et al., 2007; Suleiman et al., 2020; Jackson et al., 2021), but their model was based on data from phase III clinical trials with a relatively large sample size. Our study was based on a phase I b trial with a total of 48 patients in four cohorts, the small sample size in each cohort was not sufficient to accurately calculate response rates because the result might be easily influenced by random effects, so we did not use the response rate as the outcome when building the model.

In order to assess the accuracy of the PASI75/95 response rates calculated by simulated PASI scores, we did the visual predictive check to compare the observed time profiles of the PASI75/90 response rates versus the model-predicted time profiles. The result showed a clear underprediction of the PASI90 response rates after day 30. We think one reason was the change of efficacy from a low dose to high dose was irregular for PASI90, as can be seen in Figure 8, the PASI90 response rate in the 150 mg cohort was higher than the 300 mg cohort at days 85 and 113, which may affect the establishment of the exposure–efficacy relationship in the high-dose group. Another reason is that, one subject in the 450 mg group was excluded due to delayed administration; there were only eight subjects in this group. Due to such a small sample size, whether one subject reached PASI90 might have had a very large influence to the response rate. More data are needed to explain the uncertainity of the model in the PASI90 prediction at high-dose levels. Finally, the simulation results showed the initial phase using different loading–dosing regimens did not appear to change the onset time significantly. Administration of Q6W showed similar PASI75 and PASI90 response rates compared with Q4W in the maintenance period. This needs to be verified by future clinical studies.

The study had some limitations. Firstly, the number of patients was limited as the data were from a phase I b clinical trials. The large interindividual variability of some parameters might be due to the small sample size. Secondly, the simulation result was based on the typical value and interindividual variability estimated from 47 patients. They did not fully represent the real-world clinical setting. Thirdly, the prediction of PASI90 in the high-dose level was relatively poor. More data from further studies should be integrated to determine a better dose–response relationship.

## Conclusion

The population PK/PD model of AK111 was first established in Chinese moderate-to-severe plaque psoriasis patients based on the data of a phase Ib study. The relationship between exposure and PASI score was well described by the population model. PASI75 and PASI90 response rates of eight different dose regimens were simulated to support the dose selection and protocol design of further clinical studies.

## Data Availability

The original contributions presented in the study are included in the article/Supplementary Material; further inquiries can be directed to the corresponding authors.
